# Adhesion of streptococci to titanium and zirconia

**DOI:** 10.1371/journal.pone.0234524

**Published:** 2020-06-24

**Authors:** Yukari Oda, Tadashi Miura, Gentaro Mori, Hodaka Sasaki, Taichi Ito, Masao Yoshinari, Yasutomo Yajima

**Affiliations:** 1 Department of Oral and Maxillofacial Implantology, Tokyo Dental College, Tokyo, Japan; 2 Oral Health Science Center, Tokyo Dental College, Tokyo, Japan; Laurentian, CANADA

## Abstract

The purpose of this study was to evaluate the adherence of streptococci to disks of titanium (commercially pure titanium: CpTi) and zirconia (tetragonal zirconia polycrystals: TZP). CpTi and yttria-stabilized TZP disks with a mirror-polished surface were used as specimens. The arithmetic mean surface roughness (Ra and Sa) and the surface wettability of the experimental specimens were measured. For analyzing the outermost layer of the experimental specimens, X-ray photoelectron spectroscopy (XPS) analysis was performed. *Streptococcus sanguinis*, *S*. *gordonii*, *S*. *oralis*, and *S*. *mutans* were used as streptococcal bacterial strains. These bacterial cultures were grown for 24 h on CpTi and TZP. The number of bacterial adhesions was estimated using an ATP-bioluminescent assay, and scanning electron microscope (SEM) observation of the adhered bacterial specimens was performed. No significant differences in surface roughness or wettability were found between CpTi and TZP. In XPS analyses, outermost layer of CpTi included Ti0 and Ti^4+^, and outermost layer of TZP included Zr^4+^. In the cell adhesion assay, the adherences of *S*. *sanguinis*, *S*. *gordonii*, and *S*. *oralis* to TZP were significantly lower than those to CpTi (p < 0.05); however, significant difference was not observed for *S*. *mutans* among the specimens. The adherence to CpTi and TZP of *S*. *mutans* was significantly lower than that of *S*. *sanguinis*, *S*. *gordonii*, and *S*. *oralis*. These results were confirmed by SEM. *S*. *sanguinis*, *S*. *gordonii*, and *S*. *oralis* adhered less to TZP than to CpTi, but the adherence of *S*. *mutans* was similar to both surfaces. *S*. *mutans* was less adherent compare with the other streptococci tested in those specimens.

## Introduction

Periodontal diseases are the result of an infection process in microbial colonization referred to as dental plaque. The dental plaque is primarily formed by early-colonizing bacteria (Streptococci), following which it is formed by late-colonizing pathogenic bacteria [1]. Some late-colonizing species of oral biofilms exhibit higher pathogenic potential than early-colonizing bacteria during plaque formation [2]. Dental plaque constitutes an organized structure in which microbial colonization interacts metabolically and coexists as a community, which is known as biofilm.

Peri-implantitis is defined as a pathological condition occurring in the tissues surrounding dental implants, characterized by inflammation of the peri-implant connective tissue and progressive crestal bone loss [3]. In terms of the etiology of peri-implantitis, microbial colonization is considered to be important [4]. Similar to the process of periodontal disease, it has been reported that microbial colonization on abutment or implant surfaces can cause peri-implant mucositis [5,6], and if it is not removed, it may highly cause peri-implantitis with extensive peri-implant crestal bone loss [7]. However, microbial colonization on the implant surface remains to be fully elucidated. There are many *in vitro* papers which focused on attachment of late-colonizing anaerobic bacteria in peri-implantitis [8,9]. However, the factors that cause periodontal diseases are early-colonizing bacteria which forms flora and play an important role [10]. Current understanding of early-colonizing bacteria on the implant surface remains limited.

Recently, streptococcal species, including *Streptococcus sanguinis*, *S*. *gordonii*, *S*. *oralis*, and *S*. *mutans*, were detected in subgingival and submucosal plaque samples in peri-implantitis [11,12]. It is possible that streptococci are relevant to peri-implantitis as early-colonizing bacteria on the machined abutment surface, to which attachment is difficult. However, the adherence of early-colonizing bacteria (streptococci) to the machined abutment surface remains to be fully elucidated.

Since the 1990s, Zirconia (zirconium dioxide) has been gradually used in dentistry. Compared with other ceramics, zirconia, particularly tetragonal zirconia polycrystals (TZP), exhibit superior biomechanical properties, including a high toughness of fracture and bending strength [13]. These properties enable TZP materials to withstand oral occlusal forces [14]. Therefore, TZP is currently the material of choice for ceramic dental abutments and implants, as an alternative to titanium (CpTi).

In terms of microbial colonization and biofilm formation on TZP compared with CpTi surfaces, both *in vivo* and *in vitro* studies have been performed; however, the results reported have been controversial concerning the numbers of adhered bacteria on CpTi vs. TZP [9, 15–17]. In a previous *in vivo* study, compared to CpTi abutments, TZP abutments revealed lower plaque accumulation and less BoP (bleeding on probing) [18]. However, the mechanism remains unclear. There are some papers which focused on of late-colonizing bacteria attachment to CpTi or TZP [9,15–17]. Although early-colonizing bacteria play an important role in forming flora, there are few reports about Streptococcal attachment to CpTi and TZP. To our knowledge, there are no reports of *S*. *gordonii* and few reports of *S*. *oralis*. *S*. *sanguinis* is also under discussion. Therefore, it is important to assess whether the adherence of Streptococci is dependent on the material properties of CpTi and TZP.

The purpose of this study was to evaluate the adherence of streptococci to disks of CpTi and TZP, and compare the adherence to CpTi with that to TZP.

## Material and methods

### Specimen preparation

CpTi (grade 2; Kobe Steel) and yttria-stabilized TZP (TZ-3YB-E; Tosoh) were used in this experiment. CpTi specimens measuring 13 mm in diameter and 0.5 mm in thickness were used. They ground gradually with silicon carbide paper down to 1,200 grit, following which they were polished with 3-μm diamond pastes and 0.06-μm colloidal silica. TZP specimens measuring 13 mm in diameter and 0.5 mm in thickness were also used. They were ground with 70- and 45-μm diamond disks, following which they were finely polished with 9- and 3-μm diamond pastes and 0.06-μm colloidal silica. A polishing machine (Ecomet 3; Buehler) was used. They were cleaned by ethyl acetate, acetone, and distilled water, ultrasonically. Then, the washed disks were sterilized in an autoclave (121°C, 15 min). The sterilized disks were stored for at least 24 h until use in dry conditions.

### Surface roughness

The surface roughness was characterized using a 3D measuring laser microscope (LEXT OLS4000; OLYMPUS). The arithmetic mean of the departures of the roughness profile from the mean line and defined for a profile (Ra, 2-D) was measured with measured length 4 mm and cut-off value 0.8 mm. In addition, the arithmetic mean of the departures of the roughness area from the mean plane (Sa, corresponds to 2-D Ra) was measured with measured area 645×645 μm and Gaussian filter size 80×80 μm.

Five specimens from each group were tested.

### Surface wettability

The surface wettability of the specimens was measured by a contact angle meter (Phoenix α; Meiwa-forces), using double distilled water. Contact angles were measured at three different locations on each of the five samples, 15 s after application of the droplets. The volume of the distilled water drop was kept at 4 μL. Five specimens from each group were tested.

### X-ray photoelectron spectroscopy (XPS) analysis

XPS analysis was carried out to determine the composition of the outermost surface and chemical shift using an X-ray photoelectron spectrometer (AXIS ULTRA; Kratos Analytical) with an X-ray source of Al Kα (monochromator), 15 kV and 15 mA to determine the intensity of Ti, O, and C for the CpTi specimen, and Zr, Y, O, and C for the TZP specimen. The binding energy of each spectrum was calibrated with a C1s of 285.0 eV.

### Bacterial strains and culture media

In terms of streptococcal species, *S*. *sanguinis* ATCC 10556 (ATCC, American Type Culture Collection), *S*. *gordonii* ATCC 10558, *S*. *oralis* ATCC 35037, and *S*. *mutans* ATCC 25175 were used. The bacteria were grown on plates containing Brain heart infusion (BHI; Sigma-Aldrich) and 1.5% agar (Wako) for plate cultures. Pre-culture was performed in an anaerobic chamber (N2: 80%; H2: 10%; CO2: 10%) at 37°C for 1 day. A single colony on the plate was cultured in BHI broth for further 24 h. A fresh culture, following liquid culture for another 4 hours, was used for the adherence experiment. The optical density of every bacterial suspension was regulated with BHI broth to 0.2 at 660 nm by a spectrophotometer (Ultrospec 2100pro; Amersham Biosciences). The bacteria for subsequent experiments were grown on disks in 24-well plates.

### Cell adhesion assay

The number of adherent bacteria to the disk surface was measured using an ATP-bioluminescent assay with a commercial kit (BacTiter-Glo Microbial Cell Viability Assay kit; Promega). Following incubation with bacteria for 24 h, all bacterial fluids on the disks were removed and washed with PBS. BacTiter-Glo reagent was applied to the disk surface at 200 μL. Following this, the activity of ATP in the solution was measured using an automatic luminometer (Gene Light Model GL-210A; Microtec Co., Ltd.), and the relative luminescence was determined. Seven specimens from each material were used and at least three experiments were performed.

### Scanning electron microscope evaluation

The streptococci were cultured as described in the cell adhesion assay above. Following culture on the disks, the specimens were fixed with 1.25% glutaraldehyde in PBS for 2 h at room temperature. Then, the specimens were washed three times with PBS, and dehydrated using a graded ethanol series (70%, 80%, 90%, 95%, and 100%). The specimens were freeze-dried, sputter-coated with Au–Pd, and observed using SEM (JSM-6340F; JEOL).

### Data analysis

The results are expressed as the mean ± standard deviation. Statistical analysis was performed using GraphPad Prism (version 6.0; GraphPad Software Inc.). The data were tested by Shapiro-Wilk test and they were parametric. The data were analyzed using the student’s t-test, and probability (p) values of <0.05 were considered statistically significant. In the cell viability assay between the materials, one-way analysis of variance, followed by Tukey’s multiple comparison test, was used.

## Results

### Surface Ra/Sa and wettability

**Fig 1:**
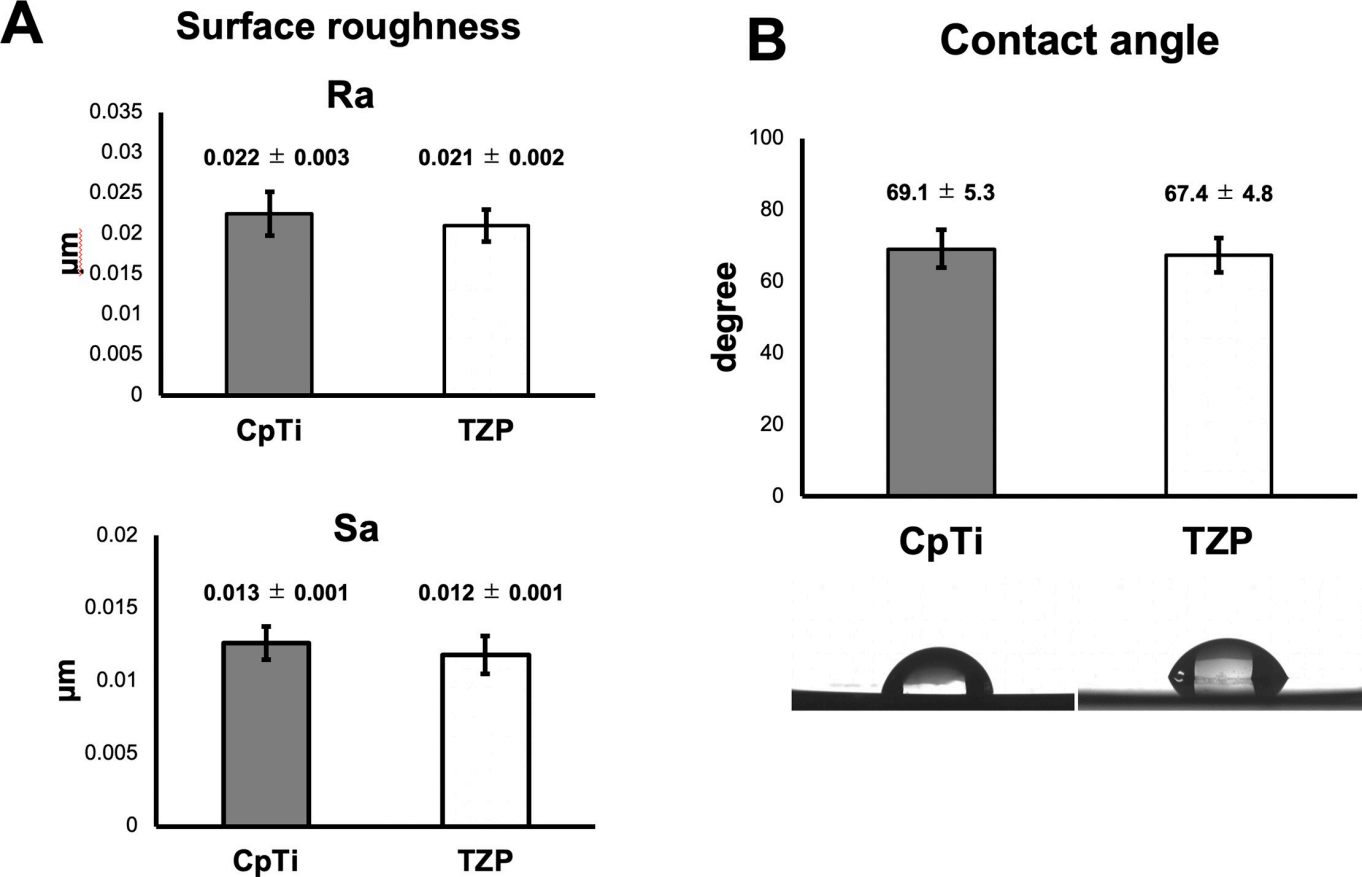
Surface Ra /Sa (A) and wettability (B). (A) The Ra and Sa values of the experimental specimens are shown. No significant differences were observed between CpTi and TZP. (B) The contact angles on the experimental specimens are shown. No clear differences were observed between CpTi and TZP.

The Ra and Sa values of the experimental specimens are shown in Fig 1A. The Ra values were 0.022 ± 0.003 μm for CpTi and 0.021 ± 0.002 μm for TZP, respectively. No significant differences were observed between CpTi and TZP. The Sa values were 0.013 ± 0.001 μm for CpTi and 0.012 ± 0.001 μm for TZP, respectively. No significant differences were observed between CpTi and TZP.

The contact angles on the experimental specimens against double distilled water and a cross-sectional view of the water droplet are shown in Fig 1B. The contact angles were 69.1 ± 5.3 degrees for CpTi and 67.4 ± 4.8 degrees for TZP. No clear differences were observed between CpTi and TZP.

### XPS analysis

**Fig 2:**
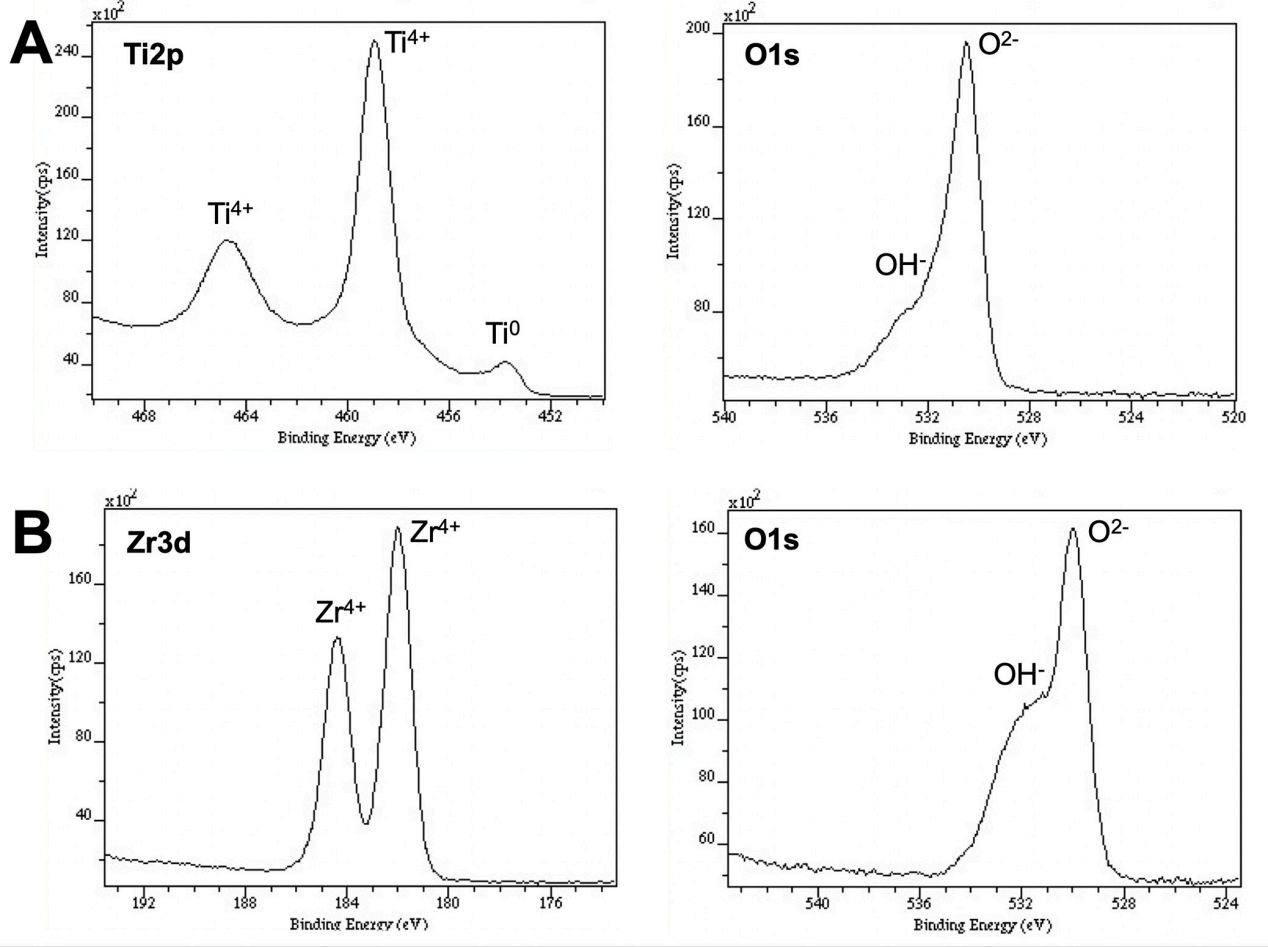
XPS analyses of the outermost layer of CpTi (A) and TZP (B). (A) The Ti2p XPS spectrum included 2p1/2 and 2p3/2 electron peaks, which decomposed into Ti0 and Ti^4+^, indicating the presence of metal and oxide, respectively. (B) The Zr3d XPS spectrum included 3d3/2 and 3d5/2 electron peaks with the presence of oxide (Zr^4+^). The O1s spectra of both CpTi and TZP have two peaks defined as TiO2 (ZrO2) at ~530.0 eV and OH at ~532.5 eV.

The results of the XPS analyses of the outermost surface of as-polished CpTi and TZP are shown in Fig 2. The Ti2p XPS spectrum included 2p1/2 and 2p3/2 electron peaks, which decomposed into Ti0 and Ti4+, indicating the presence of metal and oxide, respectively (Fig 2A). The Zr3d XPS spectrum included 3d3/2 and 3d5/2 electron peaks with the presence of oxide (Zr4+; Fig 2B). The O1s spectra of both CpTi and TZP have two peaks defined as TiO2 (ZrO2) at ~530.0 eV and OH at ~532.5 eV. In addition, a quantitative analysis showed that CpTi consisted of Ti, O, and C at concentrations of 14.8%, 44.4%, and 40.8%, respectively. TZP consisted of Z, Y, O, and C at concentrations of 13.5%, 0.7%, 40.1%, and 45.6%, respectively.

### Adhesion assay

**Fig 3:**
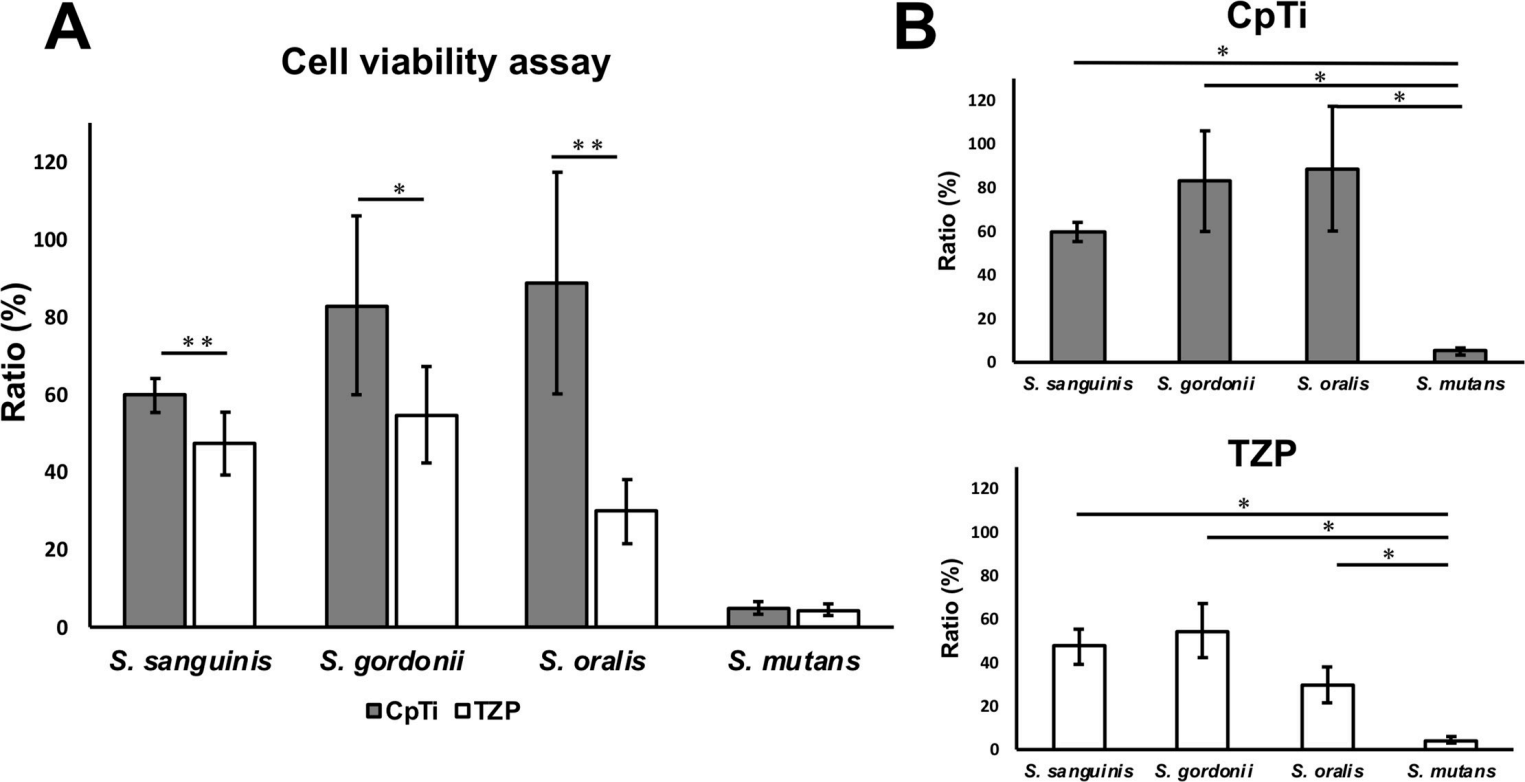
The cell viability (the relative light unit: RLU ratio) of the four streptococcal strains that adhered after 24 h. (A) The cell viability of *S*. *sanguinis*, *S*. *gordonii*, and *S*. *oralis* on TZP were significantly lower than those on CpTi. (B) On both CpTi and TZP, the cell viabilities of *S*. *sanguinis*, *S*. *gordonii*, and *S*. *oralis* were significantly higher than that of *S*. *mutans*.

The cell viability of the four streptococcal strains examined in this study that adhered after 24 h was measured by the relative light unit (RLU), as shown in Fig 3. The vertical axis “ratio (%)” shows the ratio of RLUs on the disks to the initial RLUs of the bacteria liquid cultures. The results in Fig 3A show the differences between the materials. The cell viability of *S*. *sanguinis*, *S*. *gordonii*, and *S*. *oralis* on TZP were significantly lower than those on CpTi (p < 0.05). However, no significant difference was observed in *S*. *mutans* between the specimens. The differences in bacteria are shown in Fig 3B. On both CpTi and TZP, the cell viabilities of *S*. *sanguinis*, *S*. *gordonii*, and *S*. *oralis* were significantly higher than that of *S*. *mutans* (p < 0.05).

### SEM evaluation

**Fig 4:**
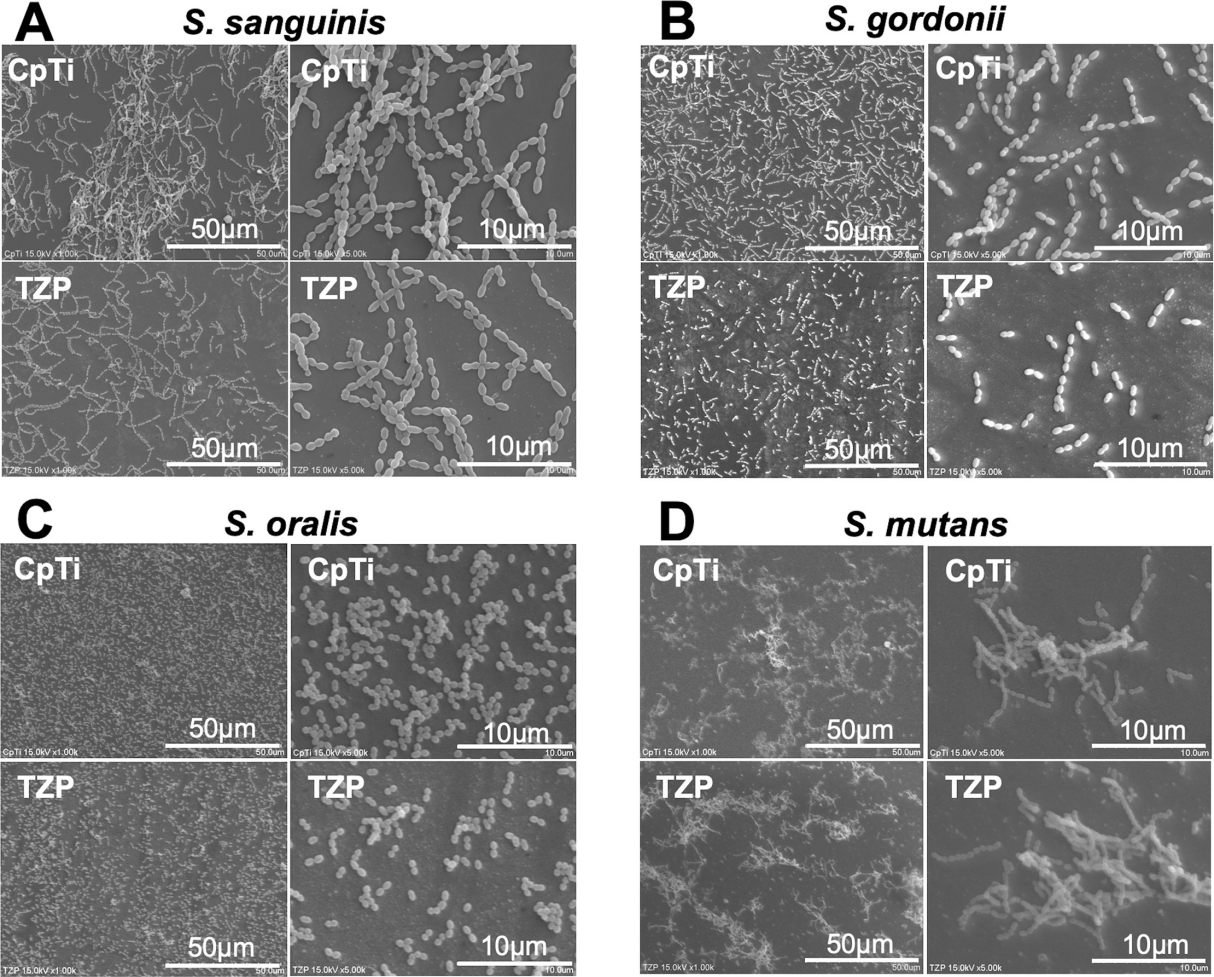
SEM images of bacteria on the CpTi and TZP surfaces. In *S*. *sanguinis*, *S*. *gordonii*, and *S*. *oralis* (A, B, C), lower bacterial adherence on TZP than on CpTi were observed. In *S*. *mutans*, no difference among specimens was observed (D).

SEM images of bacteria on the CpTi and TZP surfaces are shown in Fig 4. For *S*. *sanguinis*, *S*. *gordonii*, and *S*. *oralis*, the same findings as in the cell viability assay, that bacterial adherence on TZP was significantly lower than on CpTi, were confirmed by SEM (Fig 4A–4C). In addition, for *S*. *mutans*, the same finding as in the cell viability assay, of no difference among specimens, was confirmed (Fig 4D).

## Discussion

The present *in vitro* study investigated differences in the adherence of bacteria to CpTi and TZP. Four species of streptococci (*S*. *sanguinis*, *S*. *gordonii*, *S*. *oralis*, and *S*. *mutans*) were applied. The results indicated that *S*. *sanguinis*, *S*. *gordonii*, and *S*. *oralis* were less adherent to TZP than to CpTi, although the adherence of *S*. *mutans* to the former was similar to that to the latter. *S*. *mutans* was less adherent than the other streptococci tested in those specimens.

In material assessment, Ra were 0.021–0.022 μm for CpTi and TZP disks. It was reported that Ra of a representative commercial Ti abutment, standard Brånemark abutment, was 0.21 μm. It indicated that a reduction in surface roughness (less than a roughness of 0.2 μm) had no major effect on the bacterial attachment [19,20]. It was also reported that bacteria attached more on 3 μm gaps, however, bacteria couldn’t attach on ≤0.4 μm nano-structured gaps, which was significantly less than bacteria size [21]. In this study, Ra were 0.022 ± 0.003 μm for CpTi and 0.021 ± 0.002 μm for TZP. Thus, CpTi and TZP disks meet the requirement which is less than 0.2 μm.

In the cell adhesion and SEM assays, the adherences of *S*. *sanguinis*, *S*. *gordonii*, and *S*. *oralis* to TZP were lower than to CpTi; however, the difference was not observed in the adherence of *S*. *mutans* between the specimens. In a previous *in vitro* study, it was reported that *S*. *sanguinis* adhered more to CpTi than to TZP specimens [22]. In addition, *S*. *gordonii* and *S*. *oralis* adhered more to CpTi than to TZP specimens. Song et al [23] investigated the effects of material properties on bacterial adhesion, and reported that the hydrophobicity, roughness, topography, charges, and stiffness of a material have important effects on bacterial adhesion. In this study, there were no significant differences in surface Ra/ Sa or wettability between CpTi and TZP. This indicated that there were no significant differences in hydrophobicity, Ra and Sa, or topography between the two specimens. Accordingly, the rest charge and stiffness may affect results. In terms of charge (zeta potential), in general, most bacterial cells are charged negatively [23]. Thus, bacteria tend to adhere on a positively charged surface, whereas bacteria tend not to adhere on a negatively charged surface [23]. The isoelectric point of TiO2 is reported to be ~6.0 [24]. Accordingly, positively charged calcium ions are well adsorbed to negatively charged CpTi surfaces. This results in calcium-mediated bacterial adhesion to CpTi via CpTi/TiO2/Ca/protein/bacteria [25]. Reports on the charge (zeta potential) of TZP are limited. Zirconia is an amphoteric metal oxide that presents both anion and cation exchange properties relying on the pH and/or composition of the buffer [26]. It was also reported that the colloidal TZP powders in aqueous suspension are neutral or positively-charged [27]. Therefore, bacterial adherence to TZP can be less than that to CpTi. Further investigation is required to elucidate the correlation the electrical charge of the material surface with bacterial adherence. The influence of stiffness has received the least attention among the material properties, on which there are limited reports. Lichter et al [28] suggested that, independent of surface Ra and charge density, *S*. *epidermidis* adhesion was positively correlated with the stiffness of this material, with Young’s modulus of 0.8–80 MPa. Besides adhesion, Saha et al [29] found that *Escherichia coli* and *Lactococcus lactis* grow better on softer (Young’s modulus, 30 kPa) than harder (Young’s modulus, 150 kPa) thin polyelectrolyte multilayer films. In the present study, *S*. *sanguinis*, *S*. *gordonii*, and *S*. *oralis* may have adhered more and grown faster on CpTi (Young’s modulus, 100 GPa) than TZP (Young’s modulus, 200 GPa). As the Young’s moduli of the materials in the present study were far from those of previous reports, the direct influence of Young’s modulus may be limited. The underlying mechanism still remains unknown, however, it becomes clear that the material stiffness affects bacterial adhesion and the physiology of the adhered cells [23].

The present study found that the adherence of *S*. *mutans* to CpTi was similar to that to TZP. It has been reported that *S*. *mutans* exhibits a similar behavior in sandblasted acid-etched CpTi and machined CpTi surfaces [30].

In the comparison to each species, *S*. *mutans* was less adherent than *S*. *sanguinis*, *S*. *gordonii*, and *S*. *oralis* to the CpTi and TZP disks. There may be two reasons for this. First, in general, it is reported that there are sucrose-independent and sucrose-dependent attachments in *S*. *mutans*. A higher concentration of sucrose exposure increases adherence compared with that in the absence of sucrose for *S*. *mutans* [31]. In this study, all bacteria were cultured in BHI. This culture medium did not contain sucrose, whereas the attachment of *S*. *mutans* is sucrose-independent. This may have caused the reduced adherence of *S*. *mutans*. Second, compared with culture without disks in a 24-well plate, the adherence of *S*. *mutans* was ~20 times higher without disks than with the CpTi and TZP disks (Sup 1). Therefore, the reduced adherence may be due to the presence of the CpTi and TZP disks.

As a limitation to this study, in the culture of *S*. *mutans*, culture medium that does not contain sucrose may influence the result of the adherence assay. However, *S*. *mutans* was detected in subgingival and submucosal plaque in the peri-implantitis site,8 and the culture in this environment can be sucrose-independent.

In the present study, *S*. *sanguinis*, *S*. *gordonii*, and *S*. *oralis* were less adherent to TZP than to CpTi, although the adherence of *S*. *mutans* was similar to both CpTi and TZP. *S*. *mutans* was less adherent to CpTi and TZP compared with the other streptococcal species. It is possible that streptococci are relevant to peri-implantitis as early-colonizing bacteria on the machined TZP and CpTi abutment surfaces, to which attachment is difficult. In the future, effective prevention of peri-implantitis may be possible if it is possible to remove streptococci, the early-colonizing bacteria, as target. Further investigation is required to investigate the properties of late-colonizing pathogenic bacteria adherence following early-colonizing bacteria adherence.

## Conclusions

*S*. *sanguinis*, *S*. *gordonii*, and *S*. *oralis* were less adherent to TZP than to CpTi, although the adherence of S. mutans to TZP was similar to that to CpTi. *S*. *mutans* was less adherent compared with *S*. *sanguinis*, *S*. *gordonii*, and *S*. *oralis*.

## Supporting information

S1 Fig The adherence of *S*. *mutans*. Compared with culture without disks in a 24-well plate, the adherence of *S*. *mutans* was ~20 times higher without disks than with the CpTi and TZP disks.
(TIFF)
