# Supplementary figures and images for: Adhesion of streptococci to titanium and zirconia
Source: PLoS One. 2020 Jun 24;15(6):e0234524. doi: 10.1371/journal.pone.0234524 (PMC7314031; doi:10.1371/journal.pone.0234524)

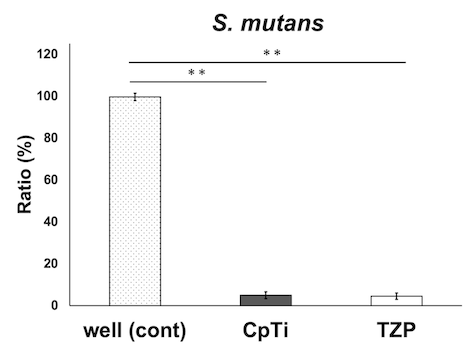

Supplement: S1 Fig — Compared with culture without disks in a 24-well plate, the adherence of S. mutans was ~20 times higher without disks than with the CpTi and TZP disks. (TIFF) [file pone.0234524.s001.tiff]
